# The advantage of flexible neuronal tunings in neural network models for motor learning

**DOI:** 10.3389/fncom.2013.00100

**Published:** 2013-07-23

**Authors:** Ellisha N. Marongelli, Kurt A. Thoroughman

**Affiliations:** Department of Biomedical Engineering, Washington University in Saint Louis, Saint LouisMO, USA

**Keywords:** motor control, generalization, neural network, computational neuroscience, motor adaptation

## Abstract

Human motor adaptation to novel environments is often modeled by a basis function network that transforms desired movement properties into estimated forces. This network employs a layer of nodes that have fixed broad tunings that generalize across the input domain. Learning is achieved by updating the weights of these nodes in response to training experience. This conventional model is unable to account for rapid flexibility observed in human spatial generalization during motor adaptation. However, added plasticity in the widths of the basis function tunings can achieve this flexibility, and several neurophysiological experiments have revealed flexibility in tunings of sensorimotor neurons. We found a model, Locally Weighted Projection Regression (LWPR), which uniquely possesses the structure of a basis function network in which both the weights and tuning widths of the nodes are updated incrementally during adaptation. We presented this LWPR model with training functions of different spatial complexities and monitored incremental updates to receptive field widths. An inverse pattern of dependence of receptive field adaptation on experienced error became evident, underlying both a relationship between generalization and complexity, and a unique behavior in which generalization always narrows after a sudden switch in environmental complexity. These results implicate a model that is flexible in both basis function widths and weights, like LWPR, as a viable alternative model for human motor adaptation that can account for previously observed plasticity in spatial generalization. This theory can be tested by using the behaviors observed in our experiments as novel hypotheses in human studies.

## Introduction

**Figure 1:**
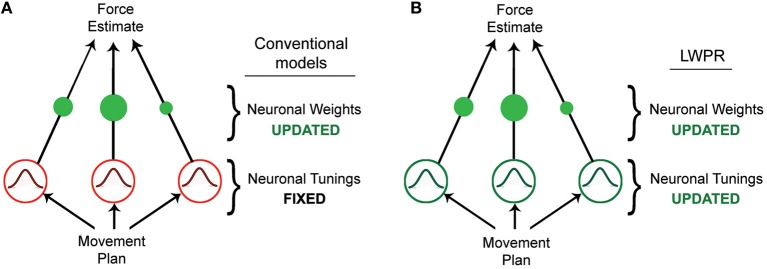
**Diagrams of different basis function network models. (A)** Diagram of a conventional basis function network model, in which learning is accomplished by incrementally updating the individual unit weights. **(B)** Diagram of the LWPR model, which possesses the same structure as a basis function network model, but in which both the weights and widths of the individual units are incrementally adapted.

Humans have the ability to skillfully adapt their movements to a variety of novel tasks and environments. Achieving this seemingly straightforward behavior, however, requires complex neurological processes and computations. Studies have shown that when learning a novel motor task, humans adapt by estimating the forces they need to exert to execute the task (Shadmehr and Mussa-Ivaldi, 1994). This force estimation is often modeled by a radial basis function neural network, in which some input is transformed via a layer of *n* nodes or neurons into an output (Pouget and Snyder, 2000; Poggio and Bizzi, 2004). In this case, the input would be properties of the desired movement, such as position, velocity, or trajectory, while the output would be the force required to execute that movement. The transforming layer is composed of a set of nodes that are tuned to the input dimensions, and whose receptive fields as a population collectively tile the input domain. By weighting these nodes (*w*_*n*_) and linearly combining their activities (*g*_*n*_), one can approximate any non-linear function *Y* of the input *x* (Figure 1A, Equation 1).

(1) $$Y=\sum w_{n}\cdot g_{n}(x)$$

The value Y is the model's prediction of the output for the given input X. The receptive fields of these nodes are generally broad Gaussian curves that respond preferentially to a particular input value. In this conventional model, the broad tunings allow for generalization across the input domain, i.e., the effect where learning in one area of the domain affects the output for different area of the domain. Wider receptive fields respond to more inputs, thereby affecting the output for a range of the domain beyond its preferred value; the Gaussian properties ensure that a receptive field's influence diminishes the further away the input is from its center. In this way, the widths of the receptive fields are inextricably related to generalization.

Learning in this model occurs via incremental adaptation of the individual node weights, usually via some gradient descent such as the delta rule (Equation 2).

(2) $$\Delta w_{n}=-a\cdot g_{n}(x_{t})(y_{t}-{\hat{y}}_{t})$$

The weights are updated by an amount that is proportional to some error signal between the actual output (*y*_*t*_) and predicted output (${\hat{y}}_{t}$), a learning rate (*a*), and the activation of their respective nodes, (*g*_*n*_) for some input *x* at time *t*, ensuring that error is minimized but that the weights are only modified when their receptive fields are appropriately relevant. Conventional implementations of this model fully attribute learning to this optimization of the weights, either implying or explicitly requiring receptive fields that are fixed in their broad shape (Poggio and Bizzi, 2004). This fixedness provides both theoretical and computational advantages for learning; it provides a consistent structural basis with which to approach novel tasks and it is more tractable to optimize weights alone instead of both weights and receptive fields' shape. However, this may be a critical limitation when trying to model human motor adaptation (Thoroughman and Taylor, 2005).

Motivated by recent observations that human generalization during motor learning is flexible (Thoroughman and Taylor, 2005), our study seeks to address the limitations of conventional motor theory and puts forth an alternative model for motor adaptation that can accommodate these observations. In the context of these psychophysical results, we test this alternative model using a simplified, but analogous experimental design to determine the mechanism and nature of generalization in the model. Our simulations generate new hypotheses that can be tested in humans to see if they employ a similar strategy when adapting their movements to novel environments.

### Flexibility in generalization

Our study is motivated by the results of a human motor adaptation experiment in which participants were directed to make horizontal reaching arm movements from a center starting position to targets in 16 radial directions while grasping a robotic manipulandum (Thoroughman and Taylor, 2005). While their arm was occluded from view, starting and target positions, as well as veridical cursor visual feedback, were provided via a projection onto a horizontal surface above their arm. Subject movements were made in the presence of viscous force fields, exerted on their arm by the robot. In order to perform the movement task correctly, participants had to successfully estimate the forces required to compensate for the ones they were experiencing through the robot. These force fields varied in spatial complexity, in that the forces were a function of the angle of movement multiplied by a complexity constant. For a simple force field, the forces changed slowly as a function of the movement angle; as the complexity increased, the forces varied more rapidly as a function of the movement angle, resulting in a more spatially dynamic force field across the workspace.

After subjects adapted to these different force fields, Thoroughman and Taylor (2005) fit the movement data to a state space model that included a function that represented the amount of generalization that was occurring across directions, i.e., how much a movement in one direction affected the updates to movement in all other directions. They observed that the amount of generalization was inversely related to the level of spatial complexity of the experienced force field. For a simple rotational field, with low spatial complexity, generalization was high and broad, extending across many directions. As field complexity increased, generalization levels became lower and less broad, with movement adaptation affecting a smaller range of directions.

This study demonstrated that under certain conditions, specifically environments of varying spatial complexity, the amount of generalization exhibited by humans and, presumably, the underlying neural structure that gives rise to generalization are indeed flexible. They showed that neural network models with different receptive field widths could achieve these differences in generalization. In the context of the conventional model for motor learning, this would imply that the receptive fields' shapes, namely their widths, are also flexible and are adapted in conjunction with the weights. This would allow for the model to optimize not what it is learning through the magnitude of the weights, but how it is learning through the generalization afforded by the receptive fields. Intuitively, this is a sensible strategy; while broad receptive fields and generalization can make learning more efficient in situations where the environment to be learned is consistently simple, the steeper slopes of narrower receptive fields allow for more sensitivity to the dynamic changes that are present in more complex environments. Lending credence to this theory are a number of examples of neurons in the visual and motor control pathways that exhibit flexibility in their tuning parameters, including preferred stimulus, slopes, and widths; we consider this physiological evidence, and the biological plausibility of our theory, in the Discussion.

Motivated by the 2005 study and neurophysiological evidence, here we assess the viability of receptive field flexibility as a critical feature in motor adaptation, something that has not yet been considered in the context of conventional learning models. We hypothesize that updating motor control theory to include this flexibility will result in improved models of human motor learning. Our experiments seek to evaluate a computational model with this structure, first to see if such a model generally exhibits a similar inverse relationship between environment complexity and generalization. Second, we want to analyze this model to identify the inherent relationships between salient learning features that facilitate these environmentally-dependent changes in generalization. These relationships can be used to form hypotheses that can be used to test for this structure in human motor studies.

### Locally weighted projection regression

We investigated several existing learning models as potential frameworks for testing the relationship between basis function width and generalization. For our computational studies, we chose to use the Locally Weighted Projection Regression (LWPR) algorithm developed by Vijayakumar et al. (2005) over other candidate models. LWPR was unique in its structure and possessed all of the key features we sought in a model for our computation studies, namely that: (1) LWPR is a monolayer radial basis function network that can model mappings between input and output parameters, (2) LWPR learns incrementally via individual training data, and (3) LWPR exhibits flexibility in both the weights and widths of its basis functions. Again, its straightforward neural network construction could be functionally relatable to neural population behavior. A detailed description of LWPR is included in the Supplementary Appendix.

Originally designed for robotics, LWPR uses a highly robust learning algorithm that is well-equipped to handle large amounts of repetitive and multi-dimensional data. The input is a target function presented as incremental training points that is transformed via a single weighted basis function layer into the predicted output (Figure 1B). The activations *g*, or receptive fields of these basis functions, are Gaussian shaped, with their width controlled by a distance metric parameter, D (Equation 3):

(3) $$g_{n}=\exp\left(-0.5{\left(x-c_{n}\right)}^{2}D_{n}\right)$$

where *x* is the input and *c*_*n*_ is the center of the receptive field. The activities of the receptive fields are linearly combined via a set of weights to produce the predicted output. The basis function *g* serves both to interpolate over the local input space, thereby avoiding overfitting, and to generate a natural domain over which individual adaptive steps affect the local and global output. This feature necessitates that even while global performance may improve and plateau over time, individual input–output training data pairs will continue to cause fluctuations about a mean in the weights at the local level (Supplementary Appendix).

Two types of error are calculated and used to update both the weights and the widths of each receptive field: the individual local error between the weighted output of each basis function and the actual output, and the global error between the overall model's predicted output and the actual output. The weights are optimized via a partial least squares regression analysis along projections in several directions in the input space, while the receptive field widths are updated using stochastic gradient descent.

Receptive fields are added or pruned locally across the input domain as needed during training to better capture the target function (Supplementary Appendix). The model adapts its widths via a gradient descent of a cost function *J* (Vijayakumar et al., 2005):

(4) $$J=\frac{1}{\sum_{i=1}^{M}w_{i}}\sum_{i=1}^{M}J_{1}+\frac{\gamma }{N}J_{2}$$

Where *M* is the number training points seen by this receptive field, *w* is the Gaussian activation of that receptive field, *N* is the dimensionality of the input domain, and γ is a tradeoff parameter. This function has two terms. The first-term calculates a proxy for mean squared error with “leave-one-out cross validation,” which avoids over-fitting to single trials. The divisor uses an inverse covariance matrix (*P* = (*X*^*T*^*WX*)^−1^)^−1^ to effectively calculate across training data without explicitly carrying all experienced errors (Schaal and Atkeson, 1998). The second term provides a penalty for inverse width such that receptive field widths are not drive to infinitesimal size (Supplementary Appendix).

There are minor implementation differences between the conventional models and LWPR. In conventional basis function networks, the population of all the weights is usually normalized such that the sum of all the weights in effect is equal to 1, so that the total contribution of all the nodes or neurons is 100 percent. Thus, the overall magnitude of the weighted prediction of each node is due to the scale of the receptive field, or the max firing rate in a neuronal context. On the contrary, in the design of LWPR, the scale of the receptive fields is normalized instead, and the magnitude comes from the weights. While the end result of the linear combination is effectively the same, as a result, our terminology conflicts with that found in the original LWPR article. As a point of clarification, the tunings that we call receptive fields the authors instead call “weights,” and the individual multipliers that we call weights the authors refer to as the individual basis function “predictions,” which is not to be confused with the overall model prediction *Y* (Equation 1). To be explicitly clear, we will continue to refer to the receptive fields as such, and to what the LWPR authors call “predictions” we will continue to refer to as weights, because we are interested primarily in the progression of the widths of the receptive fields, and not their scale.

While the neural network structure of LWPR is evident, application of the model has almost exclusively been in robotic learning, especially in biologically inspired robotic control. LWPR has been used as an effective algorithm for simulating the real-time adaptability, coordination, and robustness of human motor control in representation of the workspace and goal-oriented tasks such as reaching and grasping (Atkeson et al., 2000; Vijayakumar et al., 2002; Bendahan and Gorce, 2006; Eskiizmirliler et al., 2006; Hoffmann et al., 2007). Other studies compare LWPR performance against other real-time learning models (Nguyen-Tuong et al., 2008). However, few or no studies have directly applied the LWPR system as a model for actual biological neural computation to explain human behavior. Our experiments seek to take the fundamental mechanisms by which LWPR is a successful learning algorithm and make analogous hypotheses about real neural network features that could underlie observed human motor behavior.

## Methods

### Modifications to LWPR algorithm

**Figure 2:**
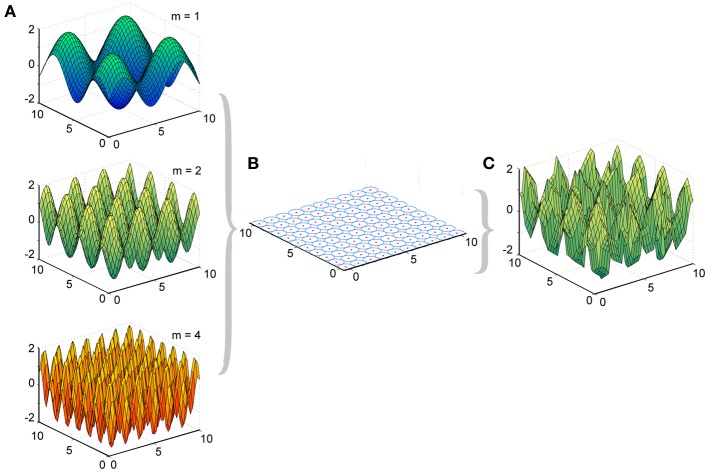
**Experimental design of LWPR target functions and basis function layout. (A)** Examples of target function sine fields of varying spatial complexities (*m* = 1, 2, and 4). **(B)** Distribution of 100 basis function receptive fields, their centers, and their widths across the input domain. **(C)** Example of a predicted function, the weighted sum of the basis functions, after LWPR learning (shown for *m* = 2).

We made two major modifications to the original LWPR MATLAB software, which was provided to us by Stefan Schaal and the Computational Learning and Motor Control Laboratory at the University of Southern California. First, our primary interest was how the receptive fields in LWPR adapted their widths. Allowing the LWPR model to add or prune units during training would provide additional higher-order mechanisms for adaptation beyond changes in receptive field widths and weights. To focus study on receptive field shapes, we disabled the online addition and pruning of receptive fields during learning. Instead, the locations of the receptive field centers were manually set by the simulation to be uniformly distributed across a 2-dimensional square input domain (Figure 2). The size of the domain to be covered and the density of the receptive fields were made adjustable parameters. This modification allowed us to better monitor the widths of the receptive fields during learning and how they individually adapted to different environments.

Second, we disabled the effect of the transient multiplier on updates to receptive field width. The purpose of the transient multiplier is to ensure that a receptive field has seen a reliable number of training points before updating its width. Because we are using a controlled source of input training functions, the transient multiplier is not required, and the receptive fields were allowed to update their widths right away during learning. Furthermore, we did not see any significant differences in final performance levels or receptive field widths when the transient multiplier was disabled. We detail these modifications in the Supplementary Appendix.

### Simulation details

We presented to the LWPR model target functions *y* that were 2-dimensional sine fields across the input domain *x* (Equation 5), where the frequency *m* controlled the complexity of the field (Figure 2).

(5) $$y=\sin(mx_{1})+\sin(mx_{2})$$

We used values of 1, 2, and 4 for *m* to simulate zero-mean 2-D fields of relatively low, medium, and high spatial complexities. Points were randomly selected without replacement from these input fields and presented as training point inputs to the model. This process was repeated as needed for more training points. To ensure consistency and reproducibility, random number seeds were reinitialized before each condition.

A population of 100 receptive fields was distributed on a uniform 10 × 10 square grid across the 2-D input domain. All receptive fields were initialized with the same width. Since the receptive fields were 2-D and elliptical, the distance metric D had the form of a 2 × 2 matrix. In our observations, the receptive fields were generally circular with little elliptical inclination, so the square root of the determinant of the D matrix was used as a singular distance metric. Because the activation functions for the receptive fields are Gaussian (Equation 2), there is no discrete measure of their width, so for the purposes of this study, we refer to a “radius” measure *r*, which is 1/v(D). The *r* for each receptive field, their individual local prediction errors, and the overall global prediction error were recorded at every training point throughout learning.

### Parameter analysis

In order to choose appropriate parameters for the model, we assessed the performance of the model while systematically varying the initial values of the two most important parameters: the distance metric D and the internal learning rate α. We tested a range of values from 10 to 100 for D and a range of values from 1e-5 to 1e4 for α at each of the three levels of spatial complexity *m* = 1, 2, and 4. For each condition, the magnitude of the global error was fitted (using least squares methods) to an exponential decay as a measure of performance over time (*t*) using three parameters for scale, decay rate, and asymptote (*k*_1_, *k*_2_, and *k*_3_ respectively; Equation 6).

(6) $$e_{\text{pred}}=k_{1}\cdot \text{exp}\left(-k_{2}t\right)+k_{3}$$

Characterizing the dependence of decay rate and asymptote on D and α permitted exploration of an operational dynamic range for these parameters and the assignment of parameters for subsequent modeling and more sophisticated analyses.

### No switch condition

Our first system analysis of the LWPR model was to observe its behavior given stable, unchanging target functions. In this condition, the input was training points derived from a single target function (*m* = 1, 2, or 4) and the model was allowed to learn this function alone until the widths of the receptive fields became relatively stable. The progression of receptive field widths throughout training was observed in detail, and compared to other salient features during learning, including individual receptive field prediction error, overall model prediction error, and individual receptive field predictions (“weights”) to identify correlative relationships that would influence how the receptive fields adapt their widths. After learning, the distributions of the receptive field widths were compared across spatial complexities. These distributions were approximately normal and were compared to each other using a *t*-test to test for significant differences in the distribution mean. To be clear, we are not using these statistical analyses for conventional hypothesis testing, but merely as a simple summary statistic to identify differences in receptive field population widths under different conditions.

### Switch condition

Finally, the other systems inspired analysis was to observe the LWPR model's behavior due to a step change in input. In this condition, the model was first presented a target function of one spatial complexity (~33,000 training points); after learning this function, the input suddenly switched to a target function of a different spatial complexity (~900,000 training points). The widths of the receptive field populations before and after the switch were assessed for trends in their adaptation behavior both qualitatively and with an ANOVA test to determine any differences between the various conditions. We also fit the magnitude of global error to exponential decays (Equation 6), one before and one after the switch, to assess any differences in learning performance due to changes in complexity.

## Results

### Parameterization

The distance metric D is the primary mechanism by which the modified LWPR algorithm adapts, being the parameter that is most flexible and incrementally optimized. This value must be carefully chosen so as to avoid local minima when optimizing receptive field widths. The internal learning rate α is a secondary mechanism by which the model constrains adaptation of the individual receptive fields. The parameter α never increases and is reduced by half only when the model is learning too fast; in this way it serves as a cap if changes in the model becomes too noisy. Therefore we sought to optimize D first, and α second.

**Figure 3:**
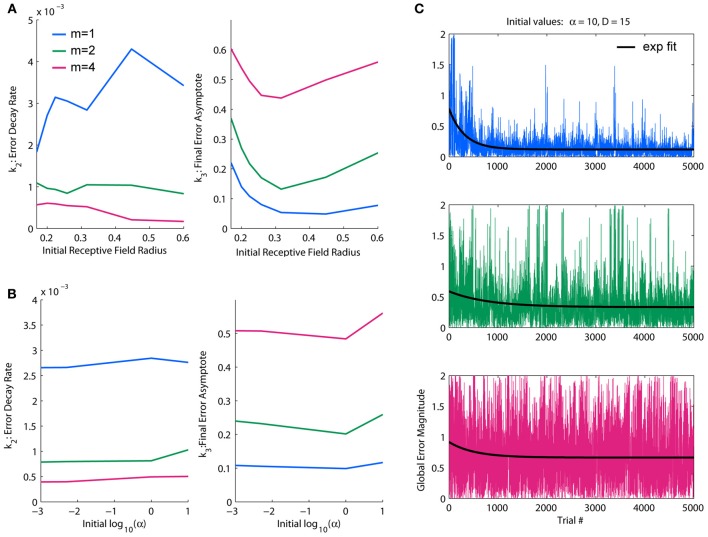
**Parameterization of LWPR initial values for learning rate and receptive field width. (A)** Effect of initial D-values on overall error decay rate and final error for spatial complexities of *m* = 1, 2, and 4. Larger initial receptive fields slow the rate of learning, but an intermediate initial width yields optimal final error levels. **(B)** Effect of initial α values on overall error decay rate and final error for spatial complexities of *m* = 1, 2, and 4. There is little effect on learning rate, but higher initial values of α worsens final error levels. **(C)** Examples of temporal error profiles and their exponential fits for each complexity condition, for initial α = 10 and initial *D* = 15. For each condition, both the global error and averaged local receptive field error is shown. Less complex functions have generally higher decay rates and lower error asymptotes than more complex functions.

We found a dynamic range of initial D values between 1 and 35, corresponding to an *r*-value range of 0.17–1; in this range, there is a clear demarcation in performance under the different complexity conditions. Fit decay rates and final error levels were averaged across different α conditions to discern general trends due to initial D-values. Fit decay rates were inversely related to complexity, while final error levels increased with complexity (Figure 3A), demonstrating that the model was able to learn lower complexity targets faster and better. Generally, decreasing the initial D-value (i.e., increasing the initial receptive field width) decreased learning rates, with effects being strongest under higher complexities. However, there was an optimal initial D-value for which the final error levels were minimized in the highest complexity condition; deviating from this value worsened performance in the highest complexity, while the other conditions were more robust to larger initial receptive field widths (Figure 3A). This is likely due to generalization (i.e., wider receptive fields) being more useful in lower complexities and more detrimental in higher complexities. We therefore chose an initial value of D near the middle of the dynamic range that optimized final performance for all conditions, *D* = 15 (*r* = 0.26).

For the observed dynamic range of D, we looked at effects of increasing orders of magnitude of α on overall learning rate and final error levels. We found the dynamic range of α to be α = 1. In this range, the relative performance levels between target functions of varying complexity were consistent with the initial D observations. The α parameter appeared to have an upper threshold of α = 1 above which final error levels increased slightly, but otherwise had little effect, especially on error decay rate or relative performance between complexities (Figure 3B). However, because a faster learning rate is theoretically more efficient and α is usually updated to be made smaller, not larger, we erred on the side of larger for the initial value, choosing one order of magnitude just at or slightly above the threshold of acceptable performance levels: α = 10. These values for D and α were used hereinafter for all simulation experiments.

An example of temporal global error profiles for each complexity condition and their fitted exponential decay functions for our chosen initial values of D and α are shown in Figure 3C. As discussed above, less complex functions generally learn faster and to lower asymptotes of error than more complex functions. While there are usually clear decays in error, we found that global error can be very noisy, with large occasional spikes. These error spikes usually occur when a training point falls in between receptive fields, i.e., when a training point fails to activate any basis function, and subsequently occurs more frequently when receptive fields are narrower. For all of our quantitative analyses, such as fitting an exponential decay to measure performance, we use global error, which is a more accurate representation of overall performance. However, in later figures regarding the switch in function complexity, which uses much longer training durations than our parameterizations, we will use moving average windows of various sizes to provide clearer visual assessments.

### Error magnitude vs. changes in receptive field widths

**Figure 4:**
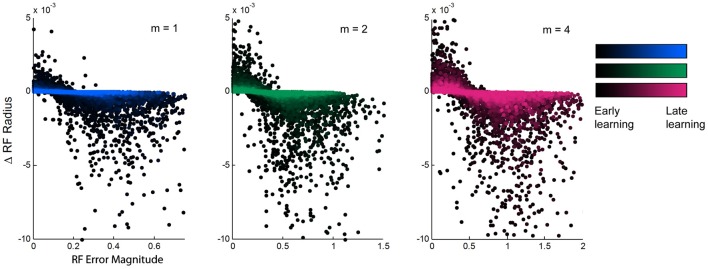
**Relationship between experienced error and receptive field adaptation during learning.** The updates to receptive field radii plotted against the magnitude of local experienced error, for spatial complexities of *m* = 1, 2, and 4, during the first ~10,000 training points of learning. There is an inverse relationship that shows receptive field narrowing when error is large and broadening with error is small. The magnitude of this effect diminishes over time from early to late learning, but its trend remains consistent.

We aimed to find a relationship between the updates to receptive field width and other features of the learning process. While we did not observe any trends due to either overall prediction error or to the individual receptive field weights, there was a clear inverse relationship between the magnitude of individual receptive field error and the subsequent update to that receptive field's width (Figure 4). When individual errors were large, the receptive fields in turn narrowed in width, presumably to increase specificity to improve performance. However, when errors were small, receptive fields did not maintain their current widths but widened. This effect is stronger in early learning than in late learning, exhibiting slopes that are much steeper early in learning that gradually flatten over time and into late learning. This shows that the model exhibits the most dramatic incremental learning early on, but the effects of individual training points diminish over time as prior experience is weighted more heavily and receptive fields converge upon stable widths. Still, the overall relationship remains consistent throughout the duration of learning, suggesting that the model is predisposed to increase generalization whenever it is affordable to do so.

### Receptive field width distributions

**Figure 5:**
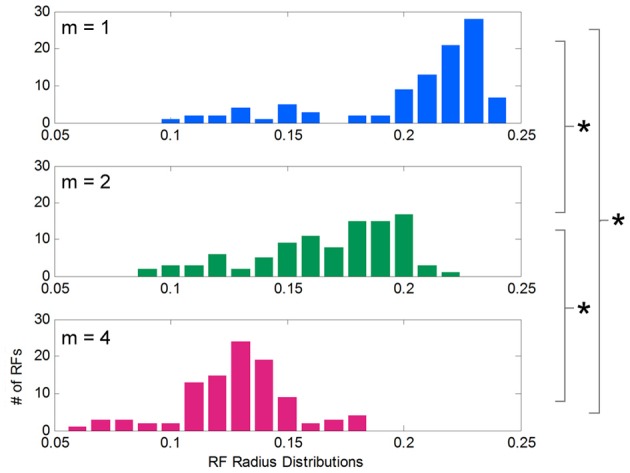
**Distribution of receptive field widths after learning functions of different spatial complexities.** Histograms showing the distribution of receptive field radii after learning target functions of varying spatial complexity. Receptive fields grow significantly narrower as spatial complexity increases.

After learning functions of low, medium, and high spatial complexity, histograms of the receptive field radii for each condition were computed and compared (Figure 5). Although all receptive fields were initialized to the same width, the distribution of adapted receptive field widths, after learning, varied with the complexity of the target function. These distributions were approximately normal in shape, and their means were inversely related to spatial complexity: for low, medium, and high complexities, the mean widths were 0.21, 0.17, and 0.13, respectively. *T*-tests showed consistent differences between these distribution means (*p* = 3.017e-17 between *m* = 1 and 2, *p* = 3.92e-26 between *m* = 2 and 4, and *p* = 1.56e-34 between *m* = 1 and 4). Low spatial complexity afforded wider receptive fields, or more generalization, while high spatial complexity induced narrower receptive fields, or less generalization. The data shown is for the chosen initial values for α and D, but we noted that this inverse relationship between adapted receptive field radii and spatial complexity was consistent across many initial values of α and D.

Here we trained our LWPR models with sine fields to most directly identify the dependence on model output and receptive field structure on spatial complexity. We also trained an LWPR model on the exact force fields used in the 2005 psychophysical study (Thoroughman and Taylor, 2005) and replicated the finding that receptive field widths narrow with increased spatial frequency (Supplemental Appendix).

### Switching spatial complexity

**Figure 6:**
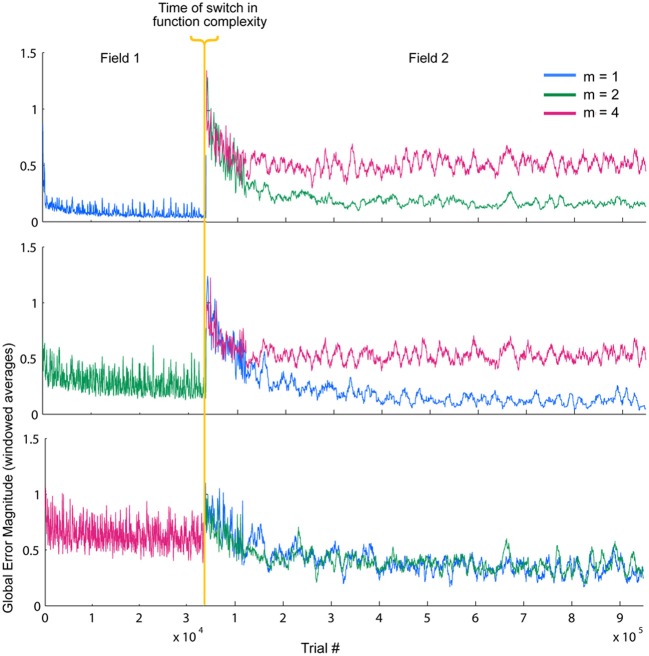
**Temporal error profiles during a switch in spatial complexity.** Global local receptive field error for each switch condition is shown, sorted by initial function complexity. To improve readability, the error has been averaged using moving window of varying sizes. The final error asymptotes after the switch are appropriately improved or worsened depending on the relative difference in function complexity before and after the switch.

So far, these results demonstrate some of the inherent relationships between function complexity, error, and receptive field widths. We now aim to assess the types of behaviors these relationships induce in the LWPR model. To do this, we examine model response when the function complexity is altered mid-training. First, fitted exponential error decays rates were always smaller after the switch. However, fitted asymptotes after the switch were appropriately improved or worsened depending on the change in relative complexity before and after the switch (Figure 6, Table 1). Switching to a more complex function resulted in a higher error asymptote, while switching to a less complex function lowered the error asymptote.

**Table 1:** **Exponential decay fits to global error magnitude before and after a switch in function complexity**.

**Field 1**			**Field 2**		
	**k2: Decay**	**k3: Asymptote**		**k2: Decay**	**k3: Asymptote**
*m* = 1	2.30E-03	0.084	*m* = 2	2.24E-05	0.241
			*m* = 4	5.65E-05	0.574
*m* = 2	1.63E-04	0.257	*m* = 4	5.18E-05	0.569
			*m* = 1	1.44E-05	0.176
*m* = 4	2.17E-04	0.620	*m* = 1	1.26E-05	0.372
			*m* = 2	1.65E-05	0.395

**Figure 7:**
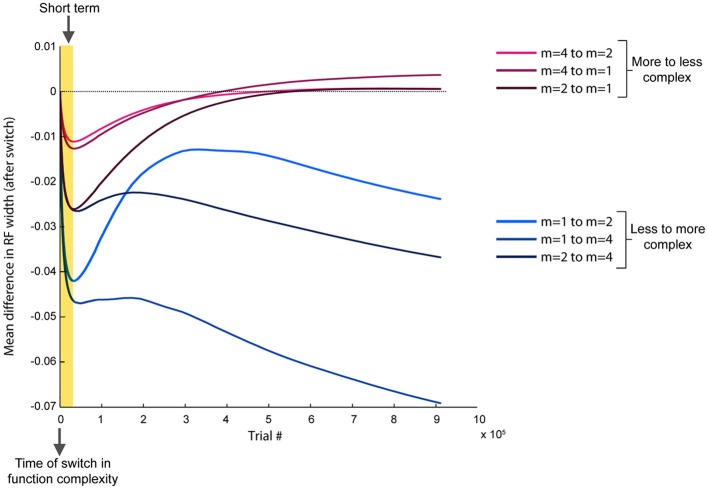
**Progression of receptive field widths after a switch in spatial complexity.** Progression of mean receptive field radii after switching the target function to a new spatial complexity, relative to the mean receptive field radii just before the switch. Short-term effects (~25,000 training points) are highlighted, while long-term effects (~900,000 training points) span the remainder of the training period. Conditions are grouped by color based on whether the new target function was more or less complex than the old target function. All conditions demonstrate a narrowing in receptive fields immediately after the switch. However, if the new function is less complex, receptive fields rebroaden, while they continue to narrow if the new function is more complex.

Based on the previously identified relationship between local error magnitude and updates to receptive field widths, we predicted that a step change in input function should initially induce a narrowing in receptive fields, due to the larger errors that would be associated with learning a new function. As the weights readjust and the LWPR model learns the new function, it should eventually adapt its receptive field widths to more appropriate values. While there was a range of adaptation behaviors for individual receptive fields due to local spatial dynamics, the overall observed mean behaviors were consistent with our hypothesis. In all conditions, shortly after the switch (~25,000 training points), there was on an average a decrease in receptive field widths compared to just before the switch (Figure 7).

However, after the model continued learning (~900,000 training points), the receptive fields adapted to a width more appropriate for the second function (Figure 7). When the switch was from a less complex to a more complex function, the receptive fields stayed narrow compared to just before the switch. However, when the switch was from a more to less complex function, the receptive fields eventually grew wider, despite having narrowed earlier just after the switch. We divided the data into two groups, more-to-less complex and less-to-more complex, each with three samples. ANOVA tests of the mean receptive field widths showed a more significant difference between the two groups in the long-term (*p* = 0.029) than in the short-term (*p* = 0.049).

## Discussion

The results of these LWPR simulations outline a succession of observations. First, this radial basis function network with both adaptable receptive field widths and weights is capable of learning functions of varying spatial complexities with a distinct, consistent strategy, in which larger errors induce narrowing of receptive fields, while smaller errors lead to widening of receptive fields. This relationship between local error magnitude and updates to receptive field widths becomes less pronounced as training progresses, but remains present throughout the duration of learning.

Secondly, this relationship with experienced error by which the widths of the receptive fields are adapted lends to the model inherently different means for learning functions of different complexities. Simpler functions tend to be easier to learn than complex functions, eliciting smaller errors. As a result, when learning simpler functions, the LWPR model ends up with relatively wide receptive fields. On the other hand, complex functions necessitated narrower, more specific receptive fields by the model. Together, these results illustrate the innate advantage of being able to generalize more under simpler spatial constraints when such broadness is economically affordable, and being able to increase the specificity of the model's underlying structure when demanded by a more complex, spatially dynamic environment.

Finally, the immediate decrease in receptive field widths following any switch in environmental complexity is a novel behavior that appears inherent to a model of this form. This unique observation is clearly an effect of the aforementioned relationship between experienced local error and updates to receptive field width; the initial spike in error caused by a sudden switch in task would intuitively induce a narrowing of receptive fields, which can only begin to approach more task-appropriate widths once performance has begun to stabilize. When conditions become more complex, long term adaptation of receptive field widths yield consistently narrower widths compared to before the switch. Conversely, when conditions become less complex, long term adaptation produces wider receptive fields. There is a clear qualitative difference in the overall trend of receptive field changes depending on the relative shift in complexity, indicating that the model behaves very differently between learning a new field that is more or less complex.

While there is a clear difference in behavior between switching to a more or less complex field, these changes are small compared to the mean receptive field widths (Figure 5). Furthermore, after switching to the new complexity, the receptive field widths never reach the same mean value that was associated with that complexity during the no-switch condition (Figure 5). In our results, when we observed that the slope of the relationship between error magnitude and receptive field updates decreased from early to late learning (Figure 4), we alluded to the fact that, like humans, LWPR is influenced by past experience. Indeed, the LWPR has a built in “forgetting factor” that controls how biased the learning is toward past experience. In addition, the implementation of the LWPR model ensures that the weights and widths of the receptive fields are converged upon gradually. The learning rate α that influences the magnitude of the updates is never increased but is reduced if changes are too large. Furthermore, projections are never removed and only added to the weight calculations if they prove to decrease error by a certain amount. These measures and the fact that receptive field properties are only updated if locally activated leads to very small updates that prevents oscillations in these values on the global timescale (Supplementary Appendix).

Thoroughman and Taylor's observation that human spatial generalization is flexible (Thoroughman and Taylor, 2005) highlights an aspect of current motor control theory that is incomplete. From their study, the psychophysical dependence of generalization on environmental complexity provided a framework by which we could similarly test the viability of LWPR as an alternative model for motor adaptation. By analyzing LWPR using target functions of varying spatial complexity, we have generated model results that can be directly tested as hypotheses in human motor adaptation studies. Thoroughman and Shadmehr demonstrated that humans similarly employ Gaussian shaped motor primitives tuned to position and velocity when estimating forces (Thoroughman and Shadmehr, 2000). If we observe in human studies that generalization narrows immediately after a switch in environment, regardless of the relative spatial complexity of the environment before and after the switch, followed by a more appropriate shift in generalization widths after longer-term learning, it would suggest that humans do employ a similar algorithm to LWPR when computing estimated forces for the task. This would first require the design of an experimental paradigm that could measure incremental changes in human motor generalization.

Since we are assuming such learning would take place in a relatively short period of time, it is a safe and practical assumption that in neurophysiology, the synaptic weights would generally not be binary (off and on), which would translate to having neural connections being completely newly forged or destroyed. Therefore, that rules out the possibility that these same end results could be computationally achieved by using a much larger population of receptive fields that have different widths but are fixed in size. Incremental optimization of the weights in this scenario would result in the weights of unfavorably sized receptive fields to completely drop out, i.e., equaling zero, so learning would otherwise have to somehow occur via a manual switch between receptive fields of different widths, which is not neurologically likely in this kind of short timescale learning.

Although LWPR advances the flexibility of model adaptation, it retains qualities of radial basis function network in that both the overall estimate (Equation 1) and the update of weights (Equation 2) linearly depend on activation. The activations in each instance, when used in motor control, are driven by the input space of trajectory kinematics. Consider a first movement A, after which weights are updated, followed by a second movement B, When the model generates a movement as updated by a single trial A, the substitution of Equation 2 into Equation 1 generates a term *g*_*n*_(*x*_*a*_)·*g*_*n*_(*x*_*b*_). The change in prediction after a single trial therefore depends on the dot product of all nodes' activity in movement A into all nodes' activity in movement B. As derived by Thoroughman and Taylor (2005), since the dependence of these activities constitute the tuning curve, the transfer of learning from movement A to movement B reveals the narrowness or width of the entire population of the underlying tuning. This algebraic finding suggests that we can seek behavioral analogs to the tuning changes in Figure 7 by measuring trial-by-trial learning transfer immediately, then eventually, following a switch in environmental complexity.

These results are especially appealing because it could have direct analogous applicability to neurophysiology. First, many neurons are tuned to position and/or velocity: in the cerebellum (Stone and Lisberger, 1990a; Coltz et al., 1999) and premotor (Johnson et al., 1999) and primary motor cortex (Georgopoulos et al., 1992; Schwartz, 1992, 1993; Ashe and Georgopoulos, 1994; Johnson et al., 1999; Moran and Schwartz, 1999; Paninski et al., 2004; Wang et al., 2007); these tunings are usually cosine or Gaussian shaped in their selectivity (Schwartz, 1993; Moran and Schwartz, 1999; Wang et al., 2007). These neurons collectively combine as a population similar to the conventional model to produce fairly accurate representations of motor output parameters (Georgopoulos et al., 1988, 1993; Schwartz, 1992, 1993; Moran and Schwartz, 1999). Furthermore, while a relationship between plasticity in these tuning curves and spatial generalization has never been explicitly addressed, there are several examples in literature of flexible neuronal tunings that correlate with improved accuracy and discriminability. These observations of flexible tunings have been mostly in visual and motor cortex, and include shifts in preferred direction (Kohn and Movshon, 2004; Ghisovan et al., 2008) and tuning curve slopes (Muller et al., 1999; Gandolfo et al., 2000; Schoups et al., 2001; Paz and Vaadia, 2004; Krekelberg et al., 2005). The authors do suggest that these changes in tuning slopes could be attributed to changes in tuning curve widths. One study that specifically investigated tuning curve widths in macaque MT found a significant trend of narrowing tuning curves during visuomotor adaptation (Krekelberg et al., 2005). These examples suggest that flexible motor bases are not only possible, but likely. In particular, Purkinje cells have been identified as potential neural analogs for the basis nodes in this model (Thoroughman and Shadmehr, 2000). These cells are located in the cerebellum, which has been recognized as a key brain area in motor coordination and adaptation processes (Hore and Flament, 1988; Baizer et al., 1999; Maschke et al., 2004; Smith and Shadmehr, 2005; Rabe et al., 2009). Purkinje cells also have broadly tuned receptive fields that respond to movement parameters (Mano and Yamamoto, 1980; Marple-Horvat, 1990; Stone and Lisberger, 1990a; Coltz et al., 1999) and receive error signals via climbing fibers (Stone and Lisberger, 1990b; Kitazawa et al., 1998), which makes them feasible candidates for basis functions in an LWPR-like motor learning model.

The results of the LWPR experiments appear to be inherent to its unique structure in which both the widths and weights of receptive fields are incrementally and concurrently adaptable. Flexible receptive fields are also consistent with physiological observations of neuronal tuning plasticity. Conventional fixed radial basis function networks cannot accommodate these findings with fixed basis functions. Even other basis function models that have mechanisms for adjusting both their weights and the shape of their receptive fields, such as Gaussian processes, are at a disadvantage because they cannot optimize both simultaneously (Rasmussen and Williams, 2006), like LWPR. In this way, LWPR stands out as a particularly viable new model for motor adaptation.

This study is an initial step in updating motor theory to better understand and represent how humans adapt their movements to novel tasks and environments. Decoding this process can elucidate human learning and motor control overall in normal subjects, leading to insights about the underlying neural processes that perform the necessary computations for motor adaptation, as well as suggest new roles for neurons throughout the brain that have been shown to exhibit flexible activity during learning.

### Conflict of interest statement

The authors declare that the research was conducted in the absence of any commercial or financial relationships that could be construed as a potential conflict of interest.
