# Supplementary material for: The advantage of flexible neuronal tunings in neural network models for motor learning
Source: Front Comput Neurosci. 2013 Jul 23;7:100. doi: 10.3389/fncom.2013.00100 (PMC3719014; doi:10.3389/fncom.2013.00100)
Supplement: Supplementary file 1 [file Presentation2.PDF]

Supplementary Appendix

The LWPR algorithm and modifications

We used the Matlab implementation of the LWPR algorithm provided by the authors at <http://www-clmc.usc.edu/software/>. Pseudocode for the complete LWPR algorithm is shown in Table 1. We modified the original code to remove certain features from the algorithm, namely the ability to add and prune receptive fields; features we have disabled are highlighted in grey.

Table 1: Modified pseudocode of the complete LWPR algorithm.
(Adapted from Vijayakumar, et al., 2005)

- Initialize the LWPR with 100 uniformly distributed receptive fields (RFs)
- For every new training sample (x,y):
  - For k = 1 to K (number of RFs)
    - * Calculate the RF activation (see Table 3)
    - * Update projections and regression (see Table 3)
    - * Update distance metric (see Table 4)
    - * Check if number of projections needs to be increased
  - If no RF was activated by more than a given threshold w_{gen},
    - * Create a new RF with R = 2, c = x, $D = init\_D$
  - If an RF significantly overlaps with another RF,
    - * Prune one of the redundant RFs

Table 2 defines a list of variables used throughout the LWPR algorithm and subsequent tables.

Table 2: Legend of indexes and symbols used for LWPR
(Adapted from Vijayakumar, et al., 2005)

Notation	Description
M	Number of training data points
N	Input dimensionality (i.e., dim. of x)
$\{x_i, y_i\}_{i=1 \ldots M}$	Training point pair, (input, output)
$k = (1 : K)$	Number of local models
$r = (1 : R)$	Number of local projections used by partial least squares regression (PLS)
$\hat{y}_k$	Local prediction (**what we call "weights"**)
$\{z_i\}_{i=1 \ldots M}$	Lower dimensional projection of input data x_i by PLS
$\{z_{i,r}\}_{r=1 \ldots R}$	Elements of projected input z_i
u_r	rth projection direction, i.e., $z_{i,r} = x_i^T u_r$
p_r	Regressed input space to be subtracted to maintain orthogonality of projection directions
w	Activation of data (x,y) on a local RF centered at c (**what we call "receptive fields")**
W^n	Sum of the weights w seen by the local RF after n data points
β_r	rth component of slope of the local linear model $\beta=[\beta_1 \ldots \beta_R]^T$
$\alpha^n_{var,r}$	Sufficient statistic for incremental computation of rth dimension of variable var after seeing n data points
M	Upper triangular matrix resulting from a Cholesky decomposition of D
P_z	Corresponds to the inverse covariance matrix computed from projected inputs z_i for $R = N$
λ	Forgetting factor
α	Learning rate that scales the magnitude of the contribution to the update
γ	Tradeoff parameter to ensure receptive fields cannot shrink indefinitely

For each receptive field, the incremental update rules for the partial least squares analysis, which optimizes that receptive field's weight, are as follows:

Table 3: Incremental locally weighted PLS for one RF centered at *c*
(Adapted from Vijayakumar, et al., 2005)

1. **Initialization:** (# data points seen $n = 0$)

   $$x_0^0 = 0,\ \ \beta_0^0 = 0,\ \ W^0 = 0,\ \ \mathrm{u}_r^0 = 0,\ \ \mathrm{p}_r^0 = 0;\ \ r = 1:R$$

2. **Incorporating new data:** Given training point (x,y)

   2a. Compute activation and update the means

   1. $w = \exp(-\tfrac{1}{2}(x-c)^T D(x-c));\quad W^{n+1} = \lambda W^n + w$
   2. $x_0^{n+1} = (\lambda W^n x_0^n + wx)/W^{n+1};\quad \beta_0^{n+1} = (\lambda W^n \beta_0^n + wy)/W^{n+1}$

   2b. Compute the current prediction error

   $x_{res,1} = x - x_0^{n+1},.\quad \hat{y} = \beta_0^{n+1}$

   Repeat for $r = 1 : R$ (# projections)

   1. $z_r = x_{res,r}^T u_r^n / \sqrt{u_r^{nT} u_r^n}$
   2. $\hat{y} \leftarrow \hat{y} + \beta_r^n z_r$
   3. $x_{res,r+1} = x_{res,r} - z_r p_r^n$
   4. $MSE_r^{n+1} = \lambda MSE_r^n + w(y-\hat{y})^2$

   $e_{cv} = y - \hat{y}$

   2c. Update the local model

   $res_1 = y - \beta_0^{n+1}$

   For $r = 1: R$ (# projections)

   2c.1 Update the local regression and compute residuals

   1. $a_{zz,r}^{n+1} = \lambda a_{zz,r}^n + w z_r^2;\quad a_{zres,r}^{n+1} = \lambda a_{zres,r}^n + w z_r res_r$
   2. $\beta_r^{n+1} = a_{zres,r}^{n+1} / a_{zz,r}^{n+1}$
   3. $res_{r+1} = res_r - z_r \beta_r^{n+1}$
   4. $\mathrm{a}_{xz,r}^{n+1} = \lambda \mathrm{a}_{xz,r}^n + w\, x_{res,r} z_r$

   2c.2 Update the projection directions

   1. $u_r^{n+1} = \lambda u_r^n + w x_{res,r} res_r$
   2. $p_r^{n+1} = \mathrm{a}_{xz,r}^{n+1} / a_{zz,r}^{n+1}$

   $e = res_{r+1}$

3. **Predicting with novel data (x_q):** Initialize $y_q = \beta_0$, $x_q = x_q - x_0$

   Repeat for $r = 1 : R$

   - $y_q \leftarrow y_q + \beta_r s_r$ where $s_r = u_r^T x_q$
   - $x_q \leftarrow x_q - s_r p_r^n$

For each receptive field, the incremental update rules for the distance metric, which is a measure of the breadth of the receptive field, are as follows:

Table 4: Increment updates to *D* for one RF Centered at *c*
(Adapted from Vijayakumar, et al., 2005)

For the current data point x*, its PLS projection* z *and activation w:*

$$J_1 = \frac{w_i (y_i - \hat{y}_i)^2}{(1 - w_i \mathrm{z}_i^T \mathrm{P}_z \mathrm{z}_i)^2}, \qquad J_2 = \sum_{i,j=1}^{N} D_{ij}^2$$

$$\text{Cost Function: } J = \frac{1}{\sum_{i=1}^{M} w_i} \sum_{i=1}^{M} J_1 + \frac{\gamma}{N} J_2$$

$$\text{Learning } D \text{ by Gradient Descent: } \mathrm{M}^{n+1} = \mathrm{M}^n - \alpha \frac{\partial J}{\partial \mathrm{M}}, \qquad D = \mathrm{M}^T \mathrm{M}$$

$$\text{Stochastic Approximation of } \frac{\partial J}{\partial \mathrm{M}}: \quad \frac{\partial J}{\partial \mathrm{M}} \approx \left(\sum_{i=1}^{M} \frac{\partial J_1}{\partial w} \right) \frac{\partial w}{\partial \mathrm{M}} + \frac{w}{W^{n+1}} \frac{\partial J^2}{\partial \mathrm{M}}$$

$$\frac{\partial w}{\partial \mathrm{M}_{kl}} = -\frac{1}{2} w (x - c)^T \frac{\partial D}{\partial \mathrm{M}_{kl}} (x - c), \qquad \frac{\partial J_2}{\partial \mathrm{M}_{kl}} = 2 \frac{\gamma}{N} \sum_{i,j=1}^{N} D_{ij} \frac{\partial D_{ij}}{\partial \mathrm{M}_{kl}}$$

$$\frac{\partial D_{ij}}{\partial \mathrm{M}_{kl}} = \mathrm{M}_{kj} \delta_{il} + \mathrm{M}_{ki} \delta_{jl}; \quad \text{where } \delta_{ij} = 1 \text{ if } i = j \text{ else } \partial_{\mathrm{ij}} = 0$$

$$\sum_{i=1}^{M} \frac{\partial J_1}{\partial w} = \frac{e_{cv}^2}{W^{n+1}} - \frac{2e}{W^{n+1}} \mathrm{q}^T \mathrm{a}_H^n - \frac{2}{W^{n+1}} \mathrm{q}^{2^T} \mathrm{a}_G^n - \frac{a_E^{n+1}}{(W^{n+1})^2}$$

$$where = \begin{bmatrix} z_1 \\ \vdots \\ z_R \end{bmatrix}, \mathrm{z}^2 = \begin{bmatrix} z_1^2 \\ \vdots \\ z_R^2 \end{bmatrix}, \mathrm{q} = \begin{bmatrix} z_1^2 / a_{zz,1}^{n+1} \\ \vdots \\ z_R^2 / a_{zz,R}^{n+1} \end{bmatrix}, \mathrm{q}^2 = \begin{bmatrix} q_1^2 \\ \vdots \\ q_R^2 \end{bmatrix}$$

$$\mathrm{a}_H^{n+1} = \lambda \mathrm{a}_H^n + \frac{w\, e_{cv} \mathrm{z}}{(1-h)}; \quad \mathrm{a}_G^{n+1} = \lambda \mathrm{a}_G^n + \frac{w^2\, e_{cv}^2 \mathrm{z}^2}{(1-h)} \quad \text{where } h = w\, \mathrm{z}^T \mathrm{q}$$

$$a_E^{n+1} = \lambda a_E^n + w\, e_{cv}^2$$

Another modification to the Matlab code that is not shown above was the disabling of the transient multiplier. The transient multiplier is used to determine whether a receptive has seen enough training points before it can reliably update its parameters. When this feature is enabled, receptive fields do not update their sizes until several data points into training; by disabling this feature, we were able to observe updates to receptive field sizes right away from the beginning of training, giving us a more complete profile of the receptive field breadth update behaviors.

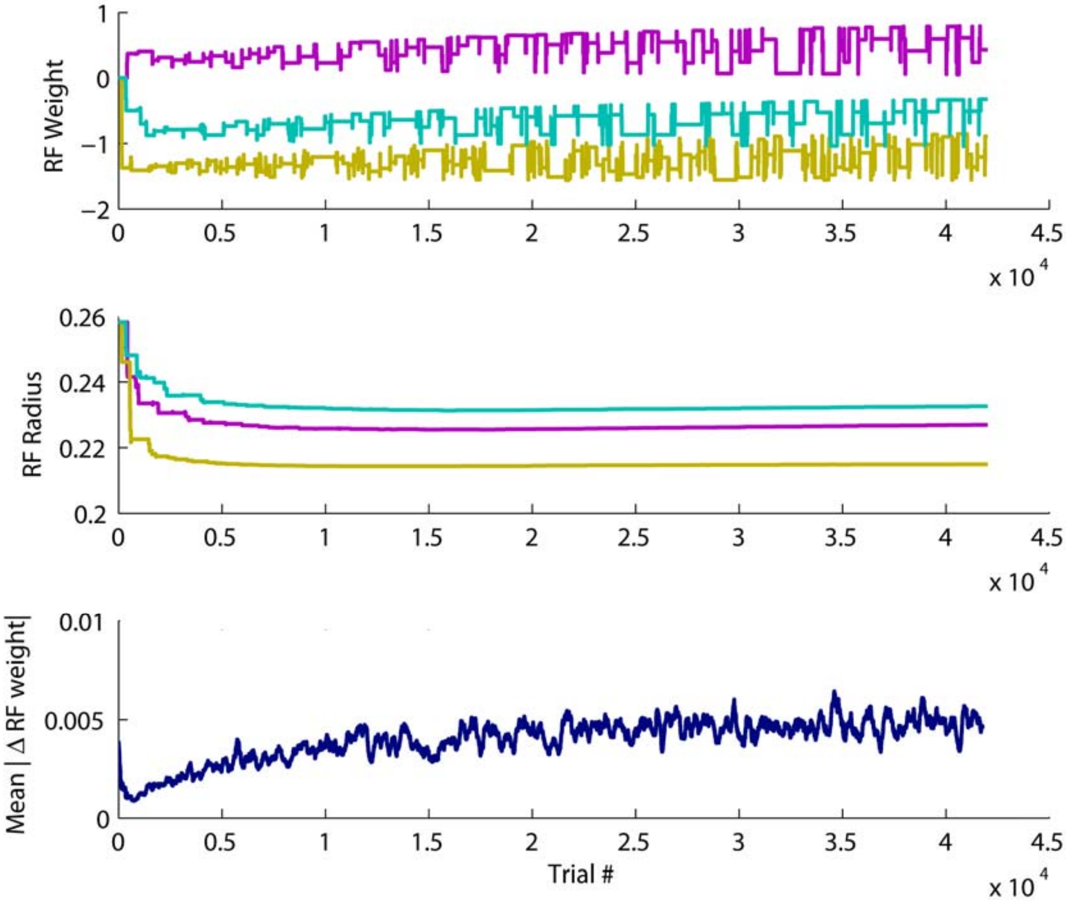

Figure 1. Updates to RF weights and widths. Incremental updates of both individual weights (*top*) and widths (*middle*) for 3 exemplar receptive fields taken from different areas of the input domain while learning a target function with m = 1. Widths are converged upon more slowly, while weights are approximated quickly and oscillate about a mean value.

(*Bottom*) Magnitude of change in weights, averaged across all 100 RFs and smoothed with a moving window of 250 trials. While the change in weights increases initially, they reach a more stable level by which the model behavior demonstrates persistent but small weight changes (updates in $\hat{y}$, Table 3, Eq. 2b.2). These changes are kept small by the dominating influence of the residual errors (Table 3, Section 2c), which decreases over time. The changes in mean RF weights exhibit a similar time series of stabilization as overall error (Manuscript Figure 6, top left).

Repeating the experiment with the force field equations from Thoroughman and Taylor, 2005

We intentionally chose to use simple sine fields for the target functions to facilitate the identification of the fundamental characteristics of learning of the LWPR model. To ensure these characteristics were consistent, we repeated the no-switch experiment with the target functions being the same equations used to generate the force fields from the Thoroughman and Taylor 2005 study [4]:

$$F = -15\sqrt{x^2 + y^2}\begin{bmatrix} -\sin(m\phi) \\ \cos(m\phi) \end{bmatrix}$$

$$\phi = \arctan\left(\frac{y}{x}\right)$$

This version had the LWPR model using a 2-D velocity input to learn a 2-D force output, as opposed to a 1-D output in our original experiment. The distribution of the 100 receptive fields was changed to span the domain area of (-5,-5) to (5,5). We found the same inverse relationship between receptive field breadths and target complexity. Mean widths were 0.199, 0.166, and 0.126, respectively. T-tests showed consistent differences between these distribution means (p = 1.89e-40 between m = 1 and 2, p = 6.56e-35 between m = 2 and 4, and p = 9.58e-51 between m = 1 and 4).

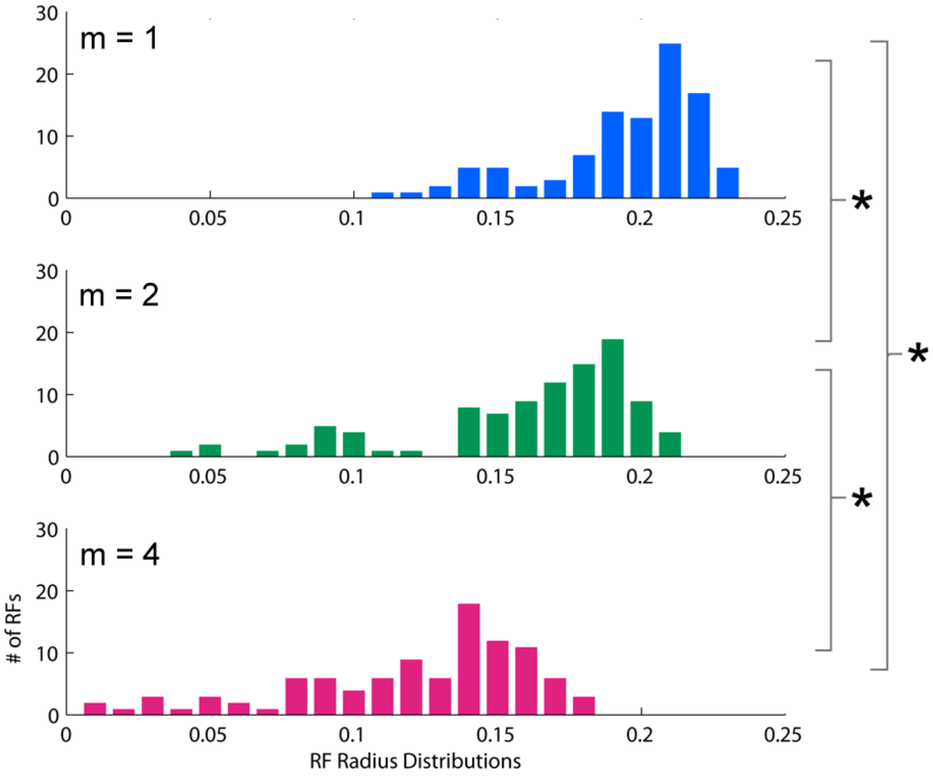

Figure 2. Distribution of receptive field sizes after learning functions of different spatial complexities. Histograms showing the distribution of receptive field radii after learning target functions of varying spatial complexity. Receptive field sizes grow significantly narrower as spatial complexity increases.
